# Hypertension in Children: Role of Obesity, Simple Carbohydrates, and Uric Acid

**DOI:** 10.3389/fpubh.2018.00129

**Published:** 2018-05-03

**Authors:** Antonina Orlando, Emanuela Cazzaniga, Marco Giussani, Paola Palestini, Simonetta Genovesi

**Affiliations:** ^1^Department of Medicine and Surgery, University of Milano-Bicocca, Milan, Italy; ^2^Family Pediatrician, Azienda Sanitaria Locale della Provincia di Milano, Milan, Italy; ^3^Department of Cardiovascular, Neural and Metabolic Sciences, S. Luca Hospital, IRCCS, Istituto Auxologico Italiano, Milan, Italy

**Keywords:** obesity, cardiovascular disease, hypertension, fructose, uric acid

## Abstract

Over the past 60 years there has been a dramatic increase in the prevalence of overweight in children and adolescents, ranging from 4% in 1975 to 18% in 2016. Recent estimates indicate that overweight or obese children and adolescents are more than 340 million. Obesity is often associated with hypertension, which is an important cardiovascular risk factor. Recent studies show that the presence of hypertension is a frequent finding in the pediatric age. Hypertensive children easily become hypertensive adults. This phenomenon contributes to increasing cardiovascular risk in adulthood. Primary hypertension is a growing problem especially in children and adolescents of western countries, largely because of its association with the ongoing obesity epidemic. Recently, it has been hypothesized that a dietary link between obesity and elevated blood pressure (BP) values could be simple carbohydrate consumption, particularly fructose, both in adults and in children. Excessive intake of fructose leads to increased serum uric acid (SUA) and high SUA values are independently associated with the presence of hypertension and weaken the efficacy of lifestyle modifications in children. The present review intends to provide an update of existing data regarding the relationship between BP, simple carbohydrates (particularly fructose), and uric acid in pediatric age. In addition, we analyze the national policies that have been carried out over the last few years, in order to identify the best practices to limit the socio-economic impact of the effects of excessive sugar consumption in children.

## Obesity: A World Disease

In the world, obesity rages like an epidemic that involves millions of people each year. The World Health Organization (WHO) has declared that around 39% of the world’s population is overweight and that worldwide obesity has nearly tripled since 1975. This alarming evidence shows that obesity is one of the major public health problems of the twenty-first century (obesity complications are the cause of about 3 million deaths per year) and the most frequent nutritional disorder in the developed countries. The infants with excess weight, in 2016, were 41 million. This problem mainly concerns the urban zone of developing countries.^1^

The prevalence of people with excess weight in Europe differs among countries: in the Mediterranean area it ranges from 20 to 40%, and in the northern countries it lies between 10 and 20% (1). These figures are alarming, especially when one considers that Mediterranean countries, which are traditionally supposed to follow a Mediterranean diet, classified as a healthy food regime, have the highest prevalence of overweight children. In 2015 in Italy, children and adolescents in excess of weight reached a proportion of 30.6%.^2^

Childhood overweight is a very common problem in high-income countries, but it has also spread in medium and low-income areas, and it is mostly due to low-quality diet and poor physical activity (2).

There are, therefore, several factors contributing to the onset of overweight; besides, genetic predisposition, socio-environmental, and psychological factors also contribute (3).

The social cost of obesity is increasing (4), but the health costs may turn out to be even higher if, on top of the cost of hospitalizations, other indirect costs attributable to obesity, such as those due to lower school performance, psychosocial problems, and poor quality of life, are added.

## Obesity and Cardiovascular Diseases (CVD)

Until a few years ago, body fat was considered an energy storage, without hormonal and metabolic functions, and its increase represented only an esthetic problem or an obstacle to physical performance, rather than a real health problem (5). Current epidemiological data show, with increasing force, that the obesity and overweight epidemic and its early onset in childhood make it necessary to consider excess weight as a cardiovascular risk factor also in pediatric age (6). Indeed, numerous studies documented an independent association between obesity and ischemic heart disease (angina and myocardial infarction) in adulthood (7). Furthermore, it has been clearly demonstrated that obesity favors heart failure, atrial fibrillation, stroke, and sudden death (7, 8). The INTERHEART study, a case–control study that looked at 29,972 patients in 52 countries, showed that the relationship between waist circumference and waist-to-hip ratio and myocardial infarction was stronger than the one between myocardial infarction and body mass index (BMI) (9). Therefore, central obesity increases the risk of developing CVD and premature deaths. Worldwide, CVD is the major cause of death and disability. In fact, in 2012 the deaths caused by CVD were 17.5 million, representing about 30% of global deaths.^3^ Even in the pediatric population, the phenomenon of obesity is widespread and, if not corrected, it can determine the onset and progression of CV complications, leading to enormous social and health costs.

## Obesity and Hypertension

Obesity is often associated with other CV risk factors such as hypertension, type 2 diabetes, endothelial dysfunction and left ventricular hypertrophy. In particular, in the pediatric population, hypertension is by far the major risk factor associated with obesity. Hypertension has been recognized globally for more than 50 years as an important risk factor for CVD in the adult population (10, 11), and its estimated prevalence is of about a half billion hypertensive subjects in 2025 (12). The literature showed that in children hypertension is not as rare as it was believed. This has led to a systematic approach to the problem in children and adolescents, with the publication of US and European recommendations on this subject (13–15). Excess weight in childhood and adolescence is the most common cause of hypertension (16–18). The first major study on pediatric hypertension stated that “the detection and management of hypertension in children and hypertension precursors in adults are the next big public health frontier” (19).

Unfortunately, to date, the diagnosis of childhood hypertension is still absent in most cases, and the knowledge of pediatric hypertension among physicians is still insufficient. The obstacles for optimum recognition of childhood hypertension include not only the limited knowledge but also the difficulty of performing multiple measurements over the years, which are essential for proper diagnosis (20, 21). In the American continent, according to the latest estimates, 74 million children under the age of 18 are hypertensive (22).

In Italy, 4% of schoolchildren have high blood pressure (BP) (16). Only 1–3% of hypertensive children are normal weight (23), while approximately 37% are overweight. An obese child is three times more at risk of developing hypertension than a normal-weight child (24).

Central obesity plays an important role in determining hypertension in the child: waist circumference, and waist-to-height ratio are in fact independent determinants of high BP beyond BMI in childhood (25). The presence of hypertension in childhood raises the probability of being hypertensive in adulthood (26).

The advances in diagnostic techniques for revealing early organ damage in the subclinical phase of hypertension have made it possible to understand that, even in pediatric age, high BP can be associated with alterations of some target organs, like left ventricular hypertrophy and increased carotid intima-media thickness (27, 28).

## Hypertension: Role of Uric Acid

In 1972, Kahn and colleagues demonstrated that increased serum uric acid (SUA) was an independent risk factor for hypertension. In particular, they found that 25–40% of adults with hypertension had SUA > 6.5 mg/dl and more than 60% had >5.5 mg/dl, and that SUA and systolic BP were linearly related. In the Multiple Risk Factor Intervention study in normotensive men, the presence of SUA levels greater than 7 mg/dl increased the risk of developing hypertension by 80%. The association between hyperuricemia and hypertension was more common in young people. High SUA was observed in about 90% of adolescents with recent onset hypertension and the SUA level correlated with BP values (29). Viazzi and colleagues found a correlation between SUA and hypertension in children with risk factors for CVD (30).

No clear causal or mechanical correlation between elevation of uric acid and hypertension development had yet been described, until in a rat model with mild hyperuricemia high uric acid levels were shown to be associated with the development of initial hypertension.

It was already known that humans and monkeys have higher SUA levels than most other mammals, due to the lack of the liver enzyme uricase that degrades uric acid to allantoin. With this knowledge, at the end of the 1990s, Johnson and colleagues developed a model of hyperuricemic rats using a pharmacological uricase inhibitor The hyperuricemic rats developed systemic hypertension, demonstrating that the increase of SUA was the cause of the increase in BP (31).

Two randomized studies showed that SUA lowering drugs were able to reduce BP values in adolescent pre-hypertensive and hypertensive individuals, demonstrating that hypertension is related with uric acid (32, 33).

Moreover, experimental studies have clarified the mechanism through which hyperuricaemia leads to increased BP. The basis of this mechanism has been hypothesized to be renal vasoconstriction that is mediated by the increase in SUA through reduction of endothelial nitric oxide release and activation of the renin–angiotensin system with consequent vascular and renal damage (34–36).

A significant association between hyperuricemia, endothelial dysfunction, and activation of the renin–angiotensin system has also been documented in humans (37, 38).

**Figure 1:**
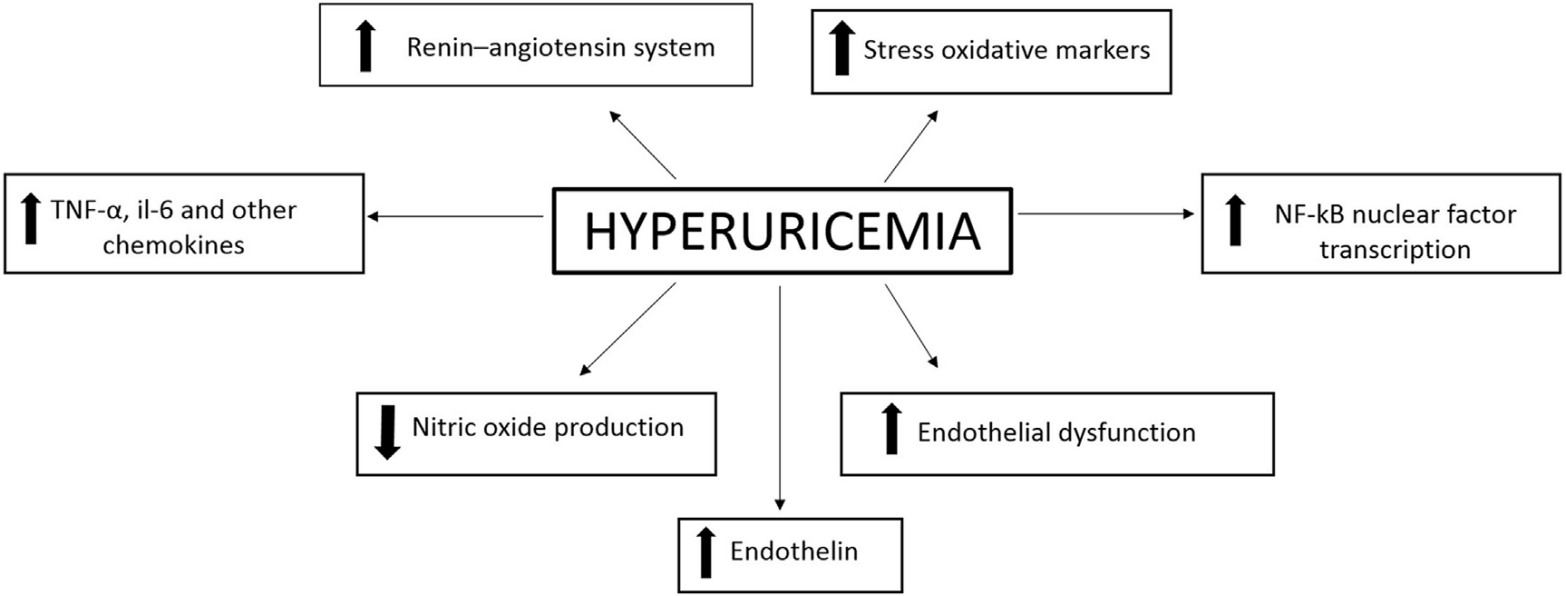
Mechanisms through which hyperuricaemia leads to cardiovascular damage.

The development of CVD may be caused by elevated values of SUA which induce inflammation at the vascular level (Figure 1).

In conclusion, all these data together suggest that uric acid promotes the development of hypertension through a two steps mechanism (39). Uric acid increases the vascular resistance by activating the renin–angiotensin–aldosterone system and suppressing the vascular nitric oxide production. Consequently, the development of the arteriosclerosis process is promoted. This process involves structural changes and is thus hardly reversible (40).

## Hypertension: Role of Fructose Consumption

The epidemic increase in hypertension and hyperuricemia may be partly due to the increase of sugar consumption (41).

Many epidemiological and experimental data have recently allowed to hypothesize that high consumption of sugar with the diet may be the mediator of the observed association between hypertension and hyperuricemia (41, 42).

**Figure 2:**
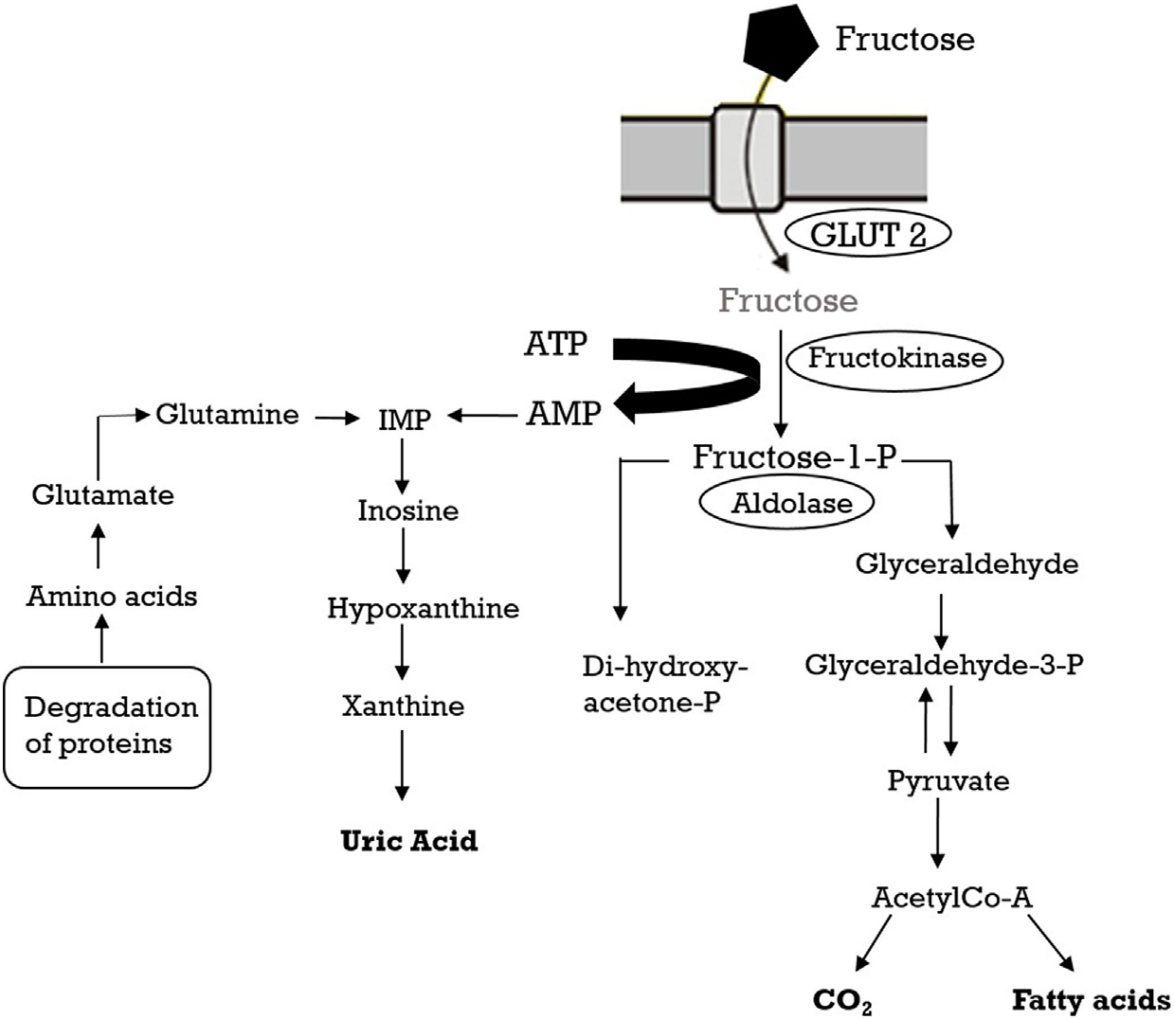
Fructose metabolism.

The consumption of added sugars, in particular fructose, and high fructose corn syrup has drastically increased (41). Excess weight and associated metabolic pathologies may be caused by the excessive intake of fructose and give rise to systemic hypertension (43). Fructose metabolism has been reviewed extensively elsewhere (44) and will be only briefly outlined here. Fructose is absorbed into the enterocytes of the small intestine by the transporter GLUT5 and successively poured into the blood by GLUT2 (45). The liver absorbs most of the fructose present in the systemic circulation and hepatic fructokinase catalyzes the phosphorylation reaction to produce fructose-1-phosphate and to initiate fructose catabolism (46). Unlike the phosphorylation of glucose by glucokinase, where a feedback system prevents excessive phosphorylation and ATP depletion, fructokinase is not inhibited by its product (fructose-1-phosphate). Consequently, the fructose metabolism leads to a fast reduction in intracellular ATP, and the AMP produced is metabolized to uric acid (Figure 2). In fact, within 30 min after fructose ingestion, a rise in uric acid can be found in the circulation (43).

Studies demonstrated that elevated fructose intake and, consequently, hyperuricemia induce inflammation in renal tubular epithelial cells leading to renal injury and development of hypertension. Indeed, drugs used to treat hyperuricemia (i.e., allopurinol) prevent this effect (47–49).

The association between fructose consumption and increase of BP levels is demonstrated by clinical and epidemiologic studies in adolescents and adults. In the National Health and Nutrition Examination Survey study population, a correlation between consumption of sugar-sweetened beverages (SSB) and higher levels of SUA and BP was shown (50, 51). Furthermore, Jalal and colleagues showed that allopurinol prevented the increase in BP values induced by the assumption of 200 g of fructose (52). In addition, a prospective study in healthy adults showed that a significant reduction of BP values was obtained by reducing intake by one SSB serving per day, independently of weight loss (53).

In adults, the correlation between sugar intake, excess weight, and cardiovascular risk was demonstrated by several epidemiologic studies. The consumption of SSB causes weight gain or increases the risk of overweight or obesity (54–56). These data emphasize the public health importance of reducing the intake of these beverages and other sweets.

## World Prevention Strategy

To manage the obesity epidemic, local and international administrations have implemented different national programs (57), in particular to fight childhood obesity.

It is complicated to evaluate the effectiveness of national public health programs (PHP) because of the number of people involved (58).

The success of a PHP requires a combination of synergistic and complementary actions, measures, regulations, and laws. Among the goals, increase in fruits and vegetables consumption, reduction in intake of simple sugars (in particular SSB), and increase in daily physical activity should not be missing.

A key strategy of the program could be to disseminate clear and simple information about its objectives and to provide dietary reference guidelines for the target population. Moreover, the collaboration between governments and the food industry should be encouraged in order to improve the quality of supplied food, as has been done in France during the French National Nutrition and Health Program. It should be noted, however, that some authors have shown that there is no evidence of the effectiveness or safety of these public–private partnerships (59).

To fight childhood obesity, the programs must affect the school settings. In France, a “Regulation on the Composition of School Meals and Food Safety” is provided to encourage school food service managers to offer fresh foods, quality products, and well-balanced meals as well as to take an active role in developing nutrition education and banning school vending machines (60).

Many authors (61–64) agree that schools are important for nutrition education because they give opportunities for experiential learning and are responsible for communication with the family and the wider community (3).

Few obesity intervention programs have targeted preschool-aged children. Lim et al. (64) tested the feasibility and effectiveness of the NASA MX project among South Korean children in kindergarten in order to promote early prevention of obesity. Their intervention program consisted in 4 weeks of fitness training and 2 weeks of nutrition education and they demonstrated that this PHP was feasible and resulted in favorable changes in eating behavior and nutritional knowledge among children.

Interestingly, different projects reported a reduction in sweet drinks (65), carbonate beverage (61), and SSB (66) consumption.

Currently, about 35–40 states in the US and the District of Columbia have sales taxes on sodas sold in grocery stores and in vending machines with the goal of decreasing caloric intake from nutrient poor foods. However, such small taxes seem to be ineffective in reducing the consumption of soda (67).

Unfortunately, some researchers (65, 68) reported the failure of a project regarding overweight children. In particular, the children involved continued with their bad habits, including excessive consumption of sweet foods, low fruit consumption, and sedentary behavior. On the other hand, the overweight children showed an improved knowledge of the attitudes to healthy eating and physical activity (68).

Some researchers (69, 70) launched the “Paying less for health” trials and showed that the price of food items influenced the purchase at the refectory or at the vending machine.

Schools should plan PHP for the prevention of obesity (61), in particular to teach how to reduce the consumption of SSBs in favor of clean drinking water (71, 72).

Ministries, research, and educational institutions are in the right position to contribute, each with their own skills, to prevent cardiovascular risk factors in children to reduce the incidence of CVD in adulthood because “cardiovascular prevention is a thing of children!”

## Author Contributions

AO and EC performed the literatures work and drafted of the article, MG, PP, and SG have done the work of review and correction.

## Conflict of Interest Statement

The authors declare that the research was conducted in the absence of any commercial or financial relationships that could be construed as a potential conflict of interest.

## Footnotes

^1^http://www.who.int/mediacentre/factsheets/fs311/en/.

^2^http://www.epicentro.iss.it/okkioallasalute/dati2016.asp.

^3^http://www.who.int/mediacentre/factsheets/fs355/en/.
